# Effects of Video-Based Application Versus Visual-Text Brochure Home Exercise in Patients with Nonspecific Neck Pain: A Randomized Controlled Trial

**DOI:** 10.3390/healthcare14183082

**Published:** 2026-09-19

**Authors:** Won-Deuk Kim, Jejeong Lee, DooChul Shin

**Affiliations:** 1 Department of Physical Therapy, Graduate School of Kyungnam University, Changwon 51767, Republic of Korea; 2 Department of Physical Therapy, Hallym Sacred Heart University, Chuncheon 24210, Republic of Korea; 3 Department of Physical Therapy, College of Future Convergence, Sahmyook University, Seoul 01795, Republic of Korea

**Keywords:** nonspecific neck pain, home exercise, smartphone application, upper cervical rotation, pressure pain threshold

## Abstract

**Highlights:**

**What are the main findings?**

- Greater improvements in upper cervical rotation range of motion were observed in the app-based home exercise group than in the handout-based exercise group.
- Pressure pain threshold improved more in the app-based group, whereas postural outcomes did not differ significantly between groups.

**What are the implications of the main findings?**

- Digital exercise instructions may be particularly useful for exercises requiring precise movement guidance.

**Abstract:**

**Background:** Nonspecific neck pain is commonly associated with limited cervical mobility, increased pain sensitivity, and postural changes. Although home exercise supports self-management, the relative effects of app-based and handout-based delivery remain unclear. **Objective:** This study compared video-based smartphone application and image- and text-based handout delivery on upper cervical rotation, pain sensitivity, and posture. **Methods:** In this pretest–posttest randomized controlled trial, 34 patients with nonspecific neck pain were assigned to an experimental or control group. Both groups received the same basic physical therapy and home exercise program three times weekly for 6 weeks. The experimental group used a video-based smartphone application, whereas the control group used a handout. Upper cervical rotation range of motion, upper trapezius pressure pain threshold, rounded shoulder height, and shoulder height angle were assessed before and after treatment. **Results:** Upper cervical rotation increased in both groups, with greater improvement in the experimental group. Left pressure pain threshold increased only in the experimental group, while right pressure pain threshold increased in both groups; improvements were greater in the experimental group bilaterally. Left rounded shoulder height decreased only in the experimental group and right rounded shoulder height decreased in both groups, but between-group differences were not significant. Shoulder height angle did not change significantly. **Conclusions:** Greater improvements in upper cervical rotation and bilateral pressure pain threshold were observed in the video-based app group than in the handout group.

## 1. Introduction

Nonspecific neck pain (NNP) refers to neck pain without an identifiable specific pathoanatomical cause on radiologic examination or routine medical assessment and is typically not accompanied by neurological symptoms [1,2]. NNP is common in the general population, and its lifetime prevalence has been reported to range from approximately 14% to 71% [3]. It is also characterized by frequent recurrence and chronicity, which indicates that NNP should be understood not simply as a transient symptom but as a musculoskeletal condition requiring continuous management [4,5].

NNP cannot be explained by a single structural lesion alone. Previous studies have reported that a history of neck pain, age, work intensity, and the level of social and occupational support are risk factors for NNP [6,7]. Individuals with NNP have been shown to have reduced active cervical range of motion (ROM), slower movement velocity, and impaired joint position sense compared with individuals without neck pain [8]. Reduced upper cervical ROM has also been reported in patients with NNP accompanied by headache [9]. These findings suggest that NNP is not merely a problem of pain but is also associated with cervical mobility, muscle function, posture, and movement characteristics. Accordingly, the home exercise program in this study was designed to address modifiable physical impairments associated with NNP, including restricted cervical mobility and altered cervical and scapular posture and movement. The program therefore included self-mobilization, scapular posterior tilt and wall slide exercises, and deep cervical flexor strengthening.

The management of NNP requires consideration not only of interventions provided in the clinic but also of activities, posture, work environment, and lifestyle outside the treatment setting. Studies reporting better long-term outcomes when workplace modification was combined with physical therapy compared with physical therapy alone suggest that activities and environmental factors outside the clinic are important in the management of NNP [10]. Therefore, patients with NNP require not only treatment in the clinical setting but also self-management strategies that can be continued in daily life.

Home exercise is a representative self-management strategy because it encourages active patient participation and enables repeated practice of exercise and postural management outside hospital visits [11]. Continuous self-management and repeated exercise performance are especially important in conditions such as NNP, in which recurrence and chronicity are common [12]. Home exercise programs have traditionally been provided as handouts consisting of images and text, and video-based home exercise delivery methods have recently been used [13]. However, image- and text-based handouts have limitations in conveying detailed exercise performance, including upper cervical movements. In addition, few studies have structured video-based home exercise programs by reflecting the physical risk factors of NNP.

Based on previously identified physical factors associated with NNP [14], a video-based smartphone application was developed to support the delivery of a structured home exercise program. The present study focused on upper cervical rotation ROM, pressure pain threshold (PPT), rounded shoulder height, and shoulder height angle as clinically relevant physical outcomes. However, previous studies using the cervical flexion–rotation test (CFRT) have reported that limited upper cervical mobility is an important clinical feature associated with cervicogenic headache and neck-related symptoms [15,16,17]. In particular, upper cervical rotation ROM has been suggested as a meaningful assessment variable for understanding cervical dysfunction. Upper cervical rotation ROM, pressure pain threshold, rounded shoulder height, and shoulder height angle are clinically relevant measures of cervical mobility, pain sensitivity, and postural alignment. In particular, rounded shoulder height and shoulder height angle were included to assess shoulder and scapular postural alignment as postural outcomes. Evaluating these outcomes may provide a comprehensive understanding of the effects of different home exercise delivery methods in patients with NNP.

Therefore, this study aimed to compare the effects of a conventional image- and text-based handout and a video-based smartphone application for delivering the same home exercise program on upper cervical rotation ROM, PPT, rounded shoulder height, and shoulder height angle in patients with NNP.

## 2. Materials and Methods

This study was a pretest–posttest randomized controlled trial comparing two methods of delivering the same home exercise program in patients with NNP. All measurements were performed by one independent assessor who was blinded to group allocation. This study was conducted in accordance with the Declaration of Helsinki and was approved by the Institutional Review Board of Kyungnam University (No. 1040460-A-2022-034, approval date: 2 November 2022). All participants received a detailed explanation of the purpose and procedures of the study, and written informed consent was obtained voluntarily before participation, including consent for the use of the collected data for research publication. Although IRB approval was obtained before participant recruitment, the trial was retrospectively registered with the Clinical Research Information Service of the Republic of Korea (CRIS; registration number: KCT0009462; registration date: 24 May 2024) because the requirement for prospective trial registration was not recognized at the time. The registration information was based on the study protocol approved by the IRB and was not subsequently modified based on the study results.

### 2.1. Participants

**Figure 1:**
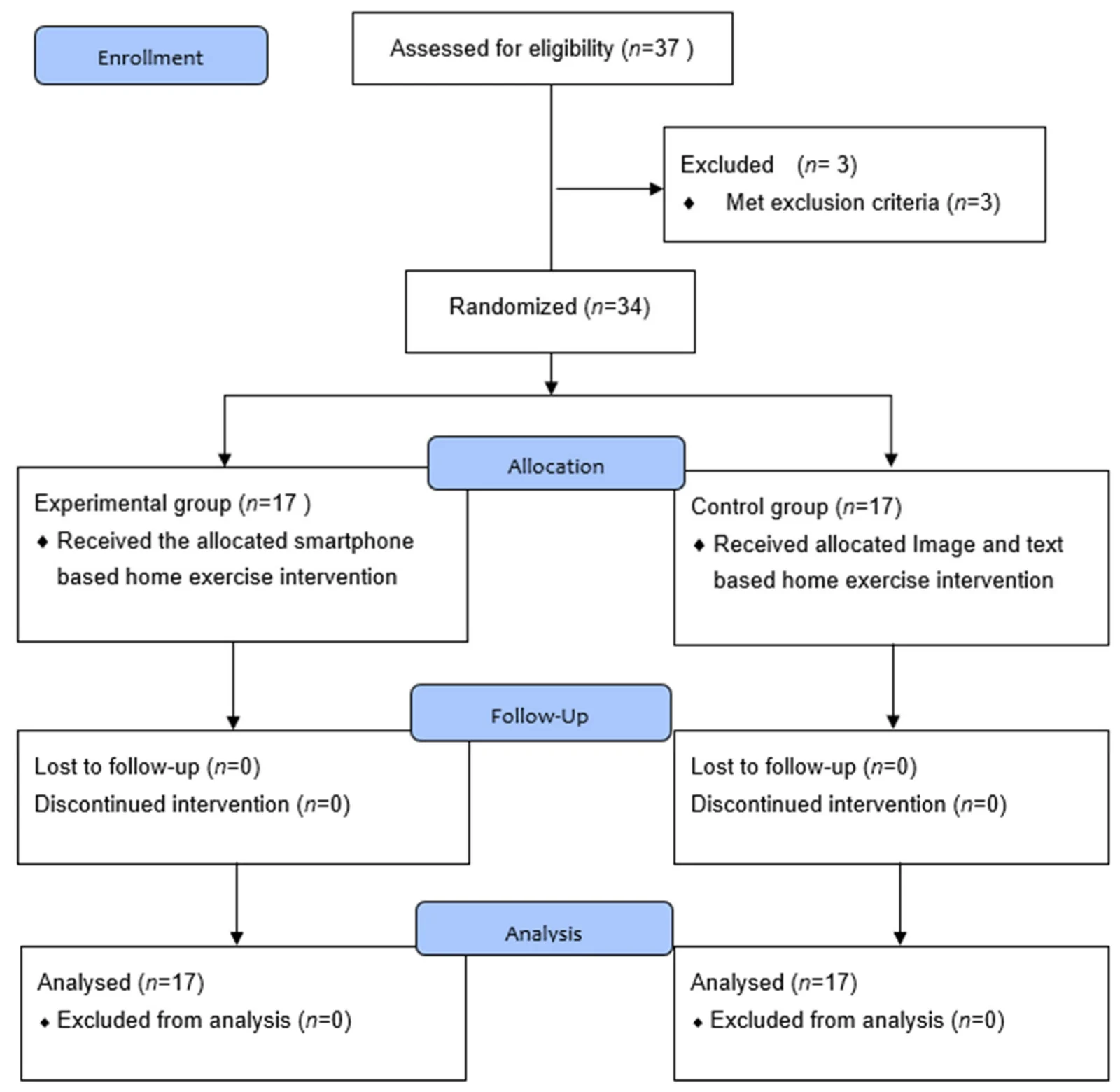
CONSORT flow diagram of the study.

An announcement was posted on the hospital bulletin board to recruit participants. Participants were recruited from patients with neck pain who visited J Hospital in Changwon, Gyeongsangnam-do, Republic of Korea. In this study, NNP was defined as pain in the neck region for which no clear pathoanatomical cause was identified on radiologic examination or medical assessment and that was not accompanied by upper extremities radiating pain or neurological symptoms. The inclusion criteria were as follows: (1) diagnosis of NNP by a physician, (2) a Numeric Pain Rating Scale score of 3 to 8 points, and (3) neck pain experienced for more than half of the time during the preceding 12 weeks. Participants who met all inclusion criteria were included in the study. Screening for the inclusion and exclusion criteria was performed by one orthopedic specialist. When necessary, additional examinations, including physical examination and magnetic resonance imaging, were performed. The exclusion criteria were as follows: (1) fracture, spinal surgery, or other orthopedic conditions, (2) central nervous system disease, such as brain injury, (3) neurological symptoms, such as numbness or burning sensation in the upper extremities, and (4) systemic inflammatory disease. The participant flow through the study is shown in Figure 1.

### 2.2. Procedures

Preintervention assessments were performed in the selected participants, and upper cervical rotation ROM, PPT, rounded shoulder height (RSH), and shoulder height difference were measured as outcome variables. The participants were then assigned to the experimental group (EG) or control group (CG) by simple randomization using a lot-drawing method. Group allocation was concealed using identical opaque lots until assignment. A research assistant generated the allocation sequence, enrolled participants, and assigned them to the intervention groups. Both groups received the same basic physical therapy and home exercise program 3 times per week for 6 weeks. The home exercise program was identical in both groups. The EG received the program through a video-based smartphone application, whereas the CG received the program through an image- and text-based handout. After the intervention, postintervention assessments were performed using the same methods as the preintervention assessments.

All preintervention and postintervention assessments were performed by one independent assessor who was unaware of the purpose of the study and the participants’ group allocation and who was not involved in the intervention process. The assessor was a physical therapist with extensive clinical and measurement experience. To reduce measurement error, the assessor completed preliminary training and standardization of all assessment procedures before the start of the study. To improve measurement reliability, all variables were measured 3 times, and the mean value was used for analysis.

### 2.3. Intervention

Both groups received the same basic physical therapy and home exercise program 3 times per week for 6 weeks. A six-week intervention period was selected because previous exercise studies in patients with neck pain have demonstrated measurable clinical changes following six weeks of repeated exercise training [18]. Basic physical therapy consisted of 20 min of hot pack therapy applied to the upper trapezius and 15 min of transcutaneous electrical nerve stimulation. Transcutaneous electrical nerve stimulation was applied in a high-frequency, low-intensity mode, with electrodes attached to the trigger point area of the upper trapezius. The same basic physical therapy protocol was applied equally to both groups.

The home exercise program was developed considering the physical risk factors of NNP. The program aimed to improve cervical and upper cervical ROM, correct rounded shoulders, correct shoulder height difference, and correct compensatory movement during cervical flexion. It included self-mobilization exercises, scapular posterior tilt exercise, wall slide exercise, and deep cervical flexor strengthening exercise.

Before beginning the home exercise program, both groups received the same face-to-face exercise instruction from the same physical therapist, with the same exercise content and level of therapist input. The EG performed the same home exercise program using a video-based smartphone application, whereas the CG performed the program using an image- and text-based handout. Therefore, the difference between the groups was not the content of the exercise program but the delivery method of the home exercise program.

### 2.4. Outcome Measures

#### 2.4.1. Upper Cervical Rotation Range of Motion

The cervical flexion–rotation test (CFRT) was performed to measure upper cervical rotation ROM. The participant was placed in the supine position, and the examiner flexed the cervical spine to the end range and then rotated the head to the right and left. The end ROM was defined as the point at which the participant reported pain or the examiner perceived a firm end feel. A digital inclinometer (Dualer IQ, JTECH Medical, Midvale, UT, USA) was placed at the center of the forehead to measure the angle. The right and left rotation ROM values were summed to calculate the total upper cervical rotation ROM used for statistical analysis. The CFRT has high test–retest reliability, with an intraclass correlation coefficient (ICC) of 0.92 [19].

#### 2.4.2. Rounded Shoulder Height

RSH was assessed by measuring the vertical distance between the table surface and the posterior aspect of the acromion in the supine position. The participant lay supine with both arms resting naturally beside the trunk, and tension around the neck and shoulders was minimized. The examiner used a ruler to measure the distance from the table surface to the posterior aspect of the acromion. A greater distance was interpreted as a more anterior position of the shoulder. This measurement method has been reported to have excellent intra-rater reliability [20].

#### 2.4.3. Shoulder Height Angle

To assess shoulder height difference, markers were attached to both acromions, and a photograph was taken. The angle between the line connecting both acromions and the horizontal line was then measured [21]. The participant stood upright with both feet shoulder-width apart and both arms resting naturally beside the trunk. Markers were attached to both acromions, and a frontal photograph was taken. The camera was fixed on a tripod and positioned parallel to the floor, with the lens height adjusted to the participant’s shoulder level. All photographs were taken using the same camera and the same angle of view, with a fixed distance of 4 m between the camera and the participant. The shoulder height angle (SHA) was measured as the angle between the line connecting both acromions and the horizontal line on the photographed image.

#### 2.4.4. Pressure Pain Threshold

PPT of the upper trapezius was measured using a digital pressure algometer (FPX25, Wagner Instruments, Greenwich, CT, USA). With the participant in a seated position, the digital pressure algometer was positioned perpendicular to the fibers of the upper trapezius, 5 to 8 cm above the superior angle of the scapula. Pressure was applied at a rate of approximately 4 to 5 N/s, and the participant was instructed to verbally report the moment when the sensation of pressure changed to pain. The contralateral upper trapezius was measured using the same procedure.

Each site was measured 3 times, and the mean value was recorded. A minimum interval of 30 s was provided between measurements. The test–retest reliability of PPT has been reported to range from ICC = 0.75 to 0.87 [22].

### 2.5. Statistical Analysis

Data were analyzed using SPSS software version 31.0 (IBM Corp., Armonk, NY, USA). General characteristics of the participants and continuous measurement variables were presented as mean and standard deviation. The distribution of the data was assessed using the Shapiro–Wilk test together with examination of skewness and kurtosis. Most variables satisfied the assumption of normality according to the Shapiro–Wilk test, although deviations from normality were observed for some variables. However, when skewness and kurtosis were also considered, no substantial violations of normality were identified. Therefore, parametric analyses were retained. Within-group differences before and after the intervention were analyzed using paired-sample *t* tests. Between-group differences in change scores (postintervention minus preintervention) were analyzed using independent-sample *t* tests. The level of statistical significance was set at α = 0.05.

## 3. Results

The first participant was enrolled on 8 November 2022, and final data collection was completed on 1 December 2023. A total of 34 participants were randomly assigned to the EG (*n* = 17) or the CG (*n* = 17), and all participants completed the study and were included in the final analysis. No intervention-related adverse events were reported. The EG included 12 men and 5 women, and the CG included 11 men and 6 women. The mean age was 41.53 years in the EG and 37.15 years in the CG. The mean height was 168.59 cm in the EG and 172.25 cm in the CG. The mean body weight was 66.94 kg in the EG and 71.65 kg in the CG. The mean pain intensity score was 4.29 points in the EG and 5.50 points in the CG. The mean disability index was 24.12% in the EG and 29.30% in the CG (Table 1).

### 3.1. Changes in Upper Cervical Rotation ROM

Upper cervical rotation ROM significantly increased in both groups, by 7.47° in the EG and by 2.95° in the CG (both *p* < 0.001). The increase was significantly greater in the EG than in the CG (*p* = 0.007) (Table 2).

### 3.2. Changes in Shoulder Height Angle

SHA decreased by 0.29° in the EG and by 0.75° in the CG. However, the within-group changes were not statistically significant in either the EG (*p* = 0.245) or the CG (*p* = 0.100). The between-group comparison of change values also showed no significant difference (*p* = 0.357) (Table 2).

### 3.3. Changes in Rounded Shoulder Height

Left RSH decreased by 3.04 cm in the EG and by 1.47 cm in the CG. The decrease was statistically significant in the EG (*p* < 0.001) but not in the CG (*p* = 0.059). Right RSH decreased by 2.84 cm in the EG and by 1.75 cm in the CG, with significant within-group changes in both the EG (*p* < 0.001) and the CG (*p* = 0.032). However, the between-group comparisons showed no significant differences in either left RSH (*p* = 0.087) or right RSH (*p* = 0.216) (Table 3).

### 3.4. Changes in PPT

Left PPT increased by 5.35 kg/cm^2^ in the EG and by 0.29 kg/cm^2^ in the CG. The increase was statistically significant in the EG (*p* = 0.035) but not in the CG (*p* = 0.680). The between-group comparison showed a significantly greater improvement in the EG than in the CG (*p* = 0.045). Right PPT increased by 6.22 kg/cm^2^ in the EG and by 1.70 kg/cm^2^ in the CG. Significant within-group increases were observed in both the EG (*p* < 0.001) and the CG (*p* = 0.021), and the improvement was significantly greater in the EG than in the CG (*p* < 0.001). The between-group effect sizes varied across outcomes, ranging from a small effect for SHA (*d* = 0.32) to a large effect for right PPT (*d* = 1.67), reflecting differences in the magnitude of between-group changes across the measured outcomes.

## 4. Discussion

This study compared the effects of delivering the same home exercise program through an image- and text-based handout or a video-based smartphone application structured according to physical risk factors in patients with NNP. The outcomes were upper cervical rotation ROM, PPT, RSH, and SHA.

Upper cervical rotation ROM significantly increased after the intervention in both the EG and CG, and the EG showed greater improvement than the CG in the between-group comparison. Upper cervical rotation represents an important component of cervical mobility, particularly because the atlantoaxial joint contributes substantially to total cervical rotation [23,24]. Reduced upper cervical mobility has also been reported in patients with neck-related symptoms [16,19]. It is difficult to determine whether reduced upper cervical mobility is a cause of pain or a consequence of pain or increased muscle tension. However, limited upper cervical ROM may reduce total cervical rotation function and contribute to compensatory movement or increased loading of the lower cervical spine or surrounding tissues, which makes it clinically meaningful. In the present study, both groups may have shown increased upper cervical ROM because the home exercise program included self-mobilization exercises targeting upper cervical rotation. In particular, this exercise requires precise control of strap or towel position, pulling direction, rotation direction, and end-range movement. Therefore, image- and text-based instructions may have limitations in fully conveying the correct performance method. The greater improvement observed in the EG may be related to the ability of the video-based application to allow participants to repeatedly check the detailed performance process of upper cervical exercises. Repeated visual demonstration may have helped participants better understand the required movement sequence and reproduce the exercise more consistently than static image- and text-based instructions. Such differences in exercise delivery may also influence movement fidelity and motor learning; however, these factors were not directly assessed in this study.

PPT increased significantly on both the left and right sides in the EG. In the CG, a significant increase was observed only on the right side, whereas the change in left PPT was not significant. Moreover, the between-group comparisons showed greater improvements in the EG for both left and right PPT. The reason for this asymmetric response remains unclear because potential side-specific factors were not assessed in this study. An increase in PPT indicates a reduction in local pain sensitivity, and PPT of the upper trapezius has been used to assess pain sensitivity and myofascial trigger-point sensitivity in patients with neck pain [22]. Exercise and patient education have also been suggested as important components for improving pain and function in patients with NNP [2]. The present findings suggest that video-based delivery may have facilitated the performance of cervical and scapular exercises and contributed to greater reductions in local pain sensitivity. However, exercise performance accuracy and adherence were not directly measured; therefore, the observed between-group differences cannot be attributed specifically to the exercise delivery format.

Left RSH significantly decreased in the EG but not in the CG, whereas right RSH significantly decreased in both groups. A decrease in RSH can be interpreted as a reduction in the anterior position of the shoulder. Rounded shoulder posture and forward head posture have been reported as postural factors associated with neck pain [25]. In addition, the acromion-to-table distance has been used as a clinical measure related to scapular anterior positioning or pectoralis minor length [20]. Side-to-side differences in scapular positioning or related soft-tissue characteristics may have contributed to the different RSH response patterns. However, these factors were not directly assessed in this study. However, no significant between-group differences were observed for either left or right RSH. Therefore, although some within-group postural changes were observed, the findings do not demonstrate that the video-based delivery method was superior to the handout-based method for improving RSH.

SHA tended to decrease in both groups, but neither the within-group changes nor the between-group comparison of change values showed significant differences. SHA is a static postural variable that reflects the relative difference in shoulder alignment between the left and right sides. It can be affected by various factors, including habitual posture, work environment, spinal and scapular alignment, and repetitive patterns of use in daily life [21]. Therefore, a 6-week home exercise program may have been insufficient to significantly change the relative difference in bilateral shoulder alignment.

Efforts were made to reduce potential bias through rigorous participant screening, assessor blinding, and the use of a single intervention provider. Additional radiologic examinations were performed when necessary to exclude participants with neurological or structural causes. In particular, this study provided a comprehensive evaluation of clinically relevant physical outcomes, including upper cervical rotation ROM, pain sensitivity, and posture-related variables, in relation to home exercise delivery methods.

This study has several limitations. A formal a priori sample-size calculation was not performed, and the sample size was small, with 17 participants in each group, which limits the generalizability of the findings. Despite randomization, baseline imbalances were observed in several outcome measures. Because unadjusted change-score analysis was used, their potential influence on the between-group differences cannot be excluded. Therefore, the between-group findings should be interpreted with caution. In addition, no adjustment for multiple comparisons was applied; therefore, the possibility of an increased Type I error should be considered when interpreting the findings, particularly those with *p* values close to the significance threshold. The intervention period was 6 weeks, and long-term effects could not be evaluated. In addition, this study did not sufficiently control for actual adherence to the home exercise program, accuracy of exercise performance, individual characteristics such as motivation and diligence, and external factors such as daily posture and work environment. Therefore, future long-term follow-up studies with larger samples are needed, and these studies should consider exercise adherence and individual characteristics related to exercise performance.

## 5. Conclusions

Greater improvements in upper cervical rotation ROM and bilateral PPT were observed in the video-based smartphone application group than in the image- and text-based handout group. Left RSH significantly decreased only in the experimental group, whereas right RSH significantly decreased in both groups; however, no significant between-group differences were observed for either side. SHA did not significantly change in either group. These findings indicate that greater improvements in upper cervical mobility and local pain sensitivity were observed in the video-based home exercise group; however, these differences cannot be attributed specifically to the delivery format because exercise adherence and performance accuracy were not measured.

## Figures and Tables

**Table 1:** General Characteristics of the Participants.

Variable	EG	CG
Gender (male/female)	12/5	11/6
Age (year)	41.53 ± 12.67	37.15 ± 10.92
Height (cm)	168.59 ± 7.78	172.25 ± 8.40
Weight (kg)	66.94 ± 13.51	71.65 ± 14.57
NRS	4.29 ± 1.49	5.5 ± 1.27
NDI (%)	24.12 ± 9.81	29.3 ± 11.56

Note. Values are presented as mean ± standard deviation. EG: Experimental group; CG: control group; NRS: Numeric Rating Scale; NDI: Neck Disability Index.

**Table 2:** Changes in Upper Cervical Rotation ROM and Shoulder Height Angle.

Variable	Group	Pre	Post	Change	*t* (*p*) ^a^	*t* (*p*) ^b^	Cohen’s *d*
UCR ROM (°)	EG	117.71 ± 5.98	125.18 ± 4.83	7.47 ± 6.15	5.01 (<0.001)	2.86 (0.007)	0.98
	CG	103.25 ± 11.10	106.20 ± 11.48	2.95 ± 2.13	5.71 (<0.001)		
SHA (°)	EG	1.76 ± 0.75	1.47 ± 0.87	−0.29 ± 0.99	1.21 (0.245)	0.94 (0.357)	0.32
	CG	2.55 ± 1.80	1.80 ± 1.00	−0.75 ± 1.77	1.75 (0.100)		

Note. Values are presented as mean ± standard deviation. ^a^ Within-group comparison. ^b^ Between-group comparison. UCR: Upper cervical rotation; ROM: range of motion; SHA: shoulder height angle; EG: experimental group; CG: control group.

**Table 3:** Changes in Rounded Shoulder Height and Pressure Pain Threshold.

Variable	Group	Pre	Post	Change	*t* (*p*) ^a^	*t* (*p*) ^b^	Cohen’s *d*
Lt. RSH (cm)	EG	6.09 ± 1.50	3.05 ± 0.89	−3.04 ± 2.13	5.88 (<0.001)	1.77 (0.087)	−0.61
	CG	9.57 ± 2.45	8.10 ± 1.48	−1.47 ± 2.98	2.03 (0.059)		
Rt. RSH (cm)	EG	6.01 ± 1.55	3.17 ± 0.80	−2.84 ± 1.81	6.47 (<0.001)	1.26 (0.216)	−0.43
	CG	9.70 ± 2.75	7.95 ± 1.43	−1.75 ± 3.07	2.35 (0.032)		
Lt. PPT (kg/cm^2^)	EG	8.70 ± 2.56	14.05 ± 1.92	5.35 ± 9.59	2.30 (0.035)	2.09 (0.045)	0.72
	CG	6.91 ± 1.92	7.20 ± 2.54	0.29 ± 2.85	0.42 (0.680)		
Rt. PPT (kg/cm^2^)	EG	8.83 ± 2.49	15.05 ± 1.47	6.22 ± 2.68	9.57 (<0.001)	4.85 (<0.001)	1.67
	CG	6.70 ± 1.79	8.40 ± 2.28	1.70 ± 2.75	2.54 (0.021)		

Note. Values are presented as mean ± standard deviation. ^a^ = Within-group comparison. ^b^ = Between-group comparison. Lt: Left; Rt: right; RSH: rounded shoulder height; PPT: pressure pain threshold.

## Data Availability

The data presented in this study are available from the corresponding author upon reasonable request. However, the data are not publicly available because public data sharing was not included in the participants’ informed consent.
